# Beyond body size—new traits for new heights in trait-based modelling of predator-prey dynamics

**DOI:** 10.1371/journal.pone.0251896

**Published:** 2022-07-21

**Authors:** Kate L. Wootton, Alva Curtsdotter, Tomas Jonsson, H. T. Banks, Riccardo Bommarco, Tomas Roslin, Amanda N. Laubmeier

**Affiliations:** 1 Swedish University of Agricultural Sciences, Department of Ecology, Uppsala, Sweden; 2 BioFrontiers Institute, University of Colorado, Boulder, Boulder, CO, United States of America; 3 Insect Ecology Lab, Zoology, The University of New England, Armidale, NSW, Australia; 4 EkoMod SpA, Comuna de Concon, Region de Valparaiso, Chile; 5 Ecological modelling group, University of Skövde, Skövde, Sweden; 6 Center for Research in Scientific Computation, North Carolina State University, Raleigh, NC, United States of America; 7 Department of Mathematics & Statistics, Texas Tech University, Lubbock, TX, United States of America; Uppsala Universitet, SWEDEN

## Abstract

Food webs map feeding interactions among species, providing a valuable tool for understanding and predicting community dynamics. Using species’ body sizes is a promising avenue for parameterizing food-web models, but such approaches have not yet been able to fully recover observed community dynamics. Such discrepancies suggest that traits other than body size also play important roles. For example, differences in species’ use of microhabitat or non-consumptive effects of intraguild predators may affect dynamics in ways not captured by body size. In Laubmeier et al. (2018), we developed a dynamic food-web model incorporating microhabitat and non-consumptive predator effects in addition to body size, and used simulations to suggest an optimal sampling design of a mesocosm experiment to test the model. Here, we perform the mesocosm experiment to generate empirical time-series of insect herbivore and predator abundance dynamics. We minimize least squares error between the model and time-series to determine parameter values of four alternative models, which differ in terms of including vs excluding microhabitat use and non-consumptive predator-predator effects. We use both statistical and expert-knowledge criteria to compare the models and find including both microhabitat use and non-consumptive predator-predator effects best explains observed aphid and predator population dynamics, followed by the model including microhabitat alone. This ranking suggests that microhabitat plays a larger role in driving population dynamics than non-consumptive predator-predator effects, although both are clearly important. Our results illustrate the importance of additional traits alongside body size in driving trophic interactions. They also point to the need to consider trophic interactions and population dynamics in a wider community context, where non-trophic impacts can dramatically modify the interplay between multiple predators and prey. Overall, we demonstrate the potential for utilizing traits beyond body size to improve trait-based models and the value of iterative cycling between theory, data and experiment to hone current insights into how traits affect food-web dynamics.

## Introduction

Mapping feeding interactions among species in food webs is a crucial first step for understanding how ecological communities function, for gauging the impacts of anthropogenic stress on community structure and stability, and for evaluating how ecosystems might be managed to conserve biodiversity and ecosystem functioning [1]. However, to achieve quantitative food-web understanding and predictions, we need a second step of formulating mechanistic models capable of replicating food-web abundance dynamics, and to develop feasible approaches to parameterize such models (e.g. [2, 3]). Only by deriving robust parameter estimates are we then prepared to predict dynamics beyond the range of the existing data, such as what happens when a new species enters the system.

Unless generalities can be identified, parameterization of a food-web model would require the strength of every trophic link to be independently estimated experimentally. Such work is both laborious and often imprecise, even for the links within a subset of a food web [4]. Additionally, some elements, such as non-consumptive interactions among multiple predators, cannot be understood solely from a pairwise predator-prey perspective because the presence of a third species modifies the interaction [5]. Although dynamic food-webs can accurately describe observed interactions, their complexity has made it unwieldy to map the abundance dynamics of diverse predator-prey assemblages in nature. Recent developments in food-web ecology are now offering a potential cure for this ‘plague of parameters’ [6] through trait-based approaches [7, 8]. Although any traits can be used in this approach, allometric (body-size based) approaches are the most common of these approaches and have been used on a wide range of species and communities [3, 9–11]. Such models assume a general relationship between organismal body size and metabolism [12, 13], and from this infer a relationship between body size and trophic interaction strength [14]. In this way, rather than estimating all parameters individually, parameters can be estimated from one easily measured trait: body size. Allometric Trophic Network (ATN) models [3, 15, 16] have been formulated based on this idea. Their results are promising, explaining a large portion of observed trophic interaction strengths and patterns of abundance dynamics of interacting species [3, 10, 11, 17], as well as replicating observed community patterns such as the mass-abundance relationship [6]. However, while body size and its effect on metabolism define, at a broad scale, how much a predator needs to eat and the size of prey it can attack and consume, many other traits can alter this relationship, leading to substantial variation unexplained by body size alone [3, 17–19]. Thus, although promising, the general applicability of body-size based models and the extent to which direct and especially indirect trophic interactions are determined by traits other than body size, remains to be explored.

Among the more successful applications of body-size based models to empirical data, Schneider et al. [3, 20] found a strong positive correlation between simulated and experimental population interaction strengths, but that the simulations overestimated the impact of spiders (who roam on the top of the litter layer) on centipedes and springtails (who predominantly dwell between litter and soil). They suggest that differences in predators’ and prey’s microhabitat use—their ‘habitat domain’ [21]—may explain the residual variation where their model did not accurately capture the experimental data. Motivated by this, Jonsson et al. [17] combined the microhabitat use of species with their body size to parameterize an extended ATN model, thereby successfully predicting experimentally observed population-level interaction strengths when a predator species was alone with its prey (i.e. in the absence of indirect effects from other species). While these and other empirical and modeling studies have pointed to the importance of predator and prey microhabitat use (e.g. [21–24]), Jonsson et al. [17] is, to our knowledge, the only study to explicitly incorporate it into a dynamic model parameterized by experimental data. They also found that increasing trophic complexity weakened the ability of their model to explain the data.

The trophic interaction modifications (or indirect trait-based effects) observed in treatments with more than two species and pinpointed by Jonsson et al. [17] are often behaviour-mediated effects on population-level interaction strengths, where changes in the behaviour of a predator and/or its prey is induced by the presence of another species, thereby modifying the per capita interaction strength between the predator and its prey [25]. Mechanisms include avoidance of intraguild predation and interference among predator species as well as facilitation [22, 26–29]. Such interactions are not described by the original ATN model, and so the model poorly captures their population-level effects [5, 17]. Jonsson et al. [17]’s results, that increasing trophic complexity weakened the ability of their model to explain the data, strongly suggested that it is a lack of behavior-based non-consumptive interspecific interference effects in the ATN model that is the main cause for its inability to accurately predict trophic interaction strength in more complex webs. Hence, two promising model developments might improve predictions: to consider the spatial niche of species and/or to account for non-consumptive intra-guild interactions.

Here we report on the findings of an experiment designed and pre-registered, but not yet run, in Laubmeier et al. [30]. Pre-registration occurs regularly in disciplines such as psychology and neurology where the research question, hypotheses and experimental procedure are developed and published a priori, to allow for feedback before the experiment begins and to prevent post-hoc alterations [31–33]. In our case, we ran pre-experimental simulations to ensure that our planned sampling design would be sufficient to obtain the data necessary to test our model. To this end, in Laubmeier et al. [30], we extended the ATN model to include new factors. We introduced a term for microhabitat use, where predators and prey will encounter each other more frequently the more time they spend in the same area, thereby showing a stronger interaction strength. In [30] we also included a term for non-consumptive intra- and interspecific predator-predator effects, where avoidance of other predators due to the fear of intraguild predation or interference by other predators decreases predation rate (e.g. [26, 34, 35]). Finally, to establish whether the effects of microhabitat use and non-consumptive predator-predator effects were sufficiently strong to be observed across a diverse range of predators, we intentionally selected predators covering a range of guilds and feeding modes. To accommodate effects of variation in feeding mode or other traits not included in the model for different types of predators, we allowed the value of the optimal predator-prey body-mass ratio to vary from predator to predator in the parameter estimation that followed. In this respect our approach departs from other studies utilizing the ATN model, such as Schneider et al. [3] and Jonsson et al. [17] where the optimal predator-prey body mass ratio was fixed across predators.

## The model

We model predator-prey population dynamics in a food web, assuming species’ interaction strengths are determined by body size and microhabitat use. Stronger interactions occur when prey are close to a predator’s optimal prey size, or when predator and prey overlap more in their microhabitat use. To develop our model, we started with the Allometric Trophic Network (ATN) model, which uses body sizes of predator and prey to dictate interaction strengths [3, 14, 15]. We then extended the ATN model to include microhabitat overlap and non-consumptive predator-predator interactions.

Our modified model was published in Laubmeier et al. [30]. Subsequent to publication, we observed that our original formulation for similarity in microhabitat use (which was also used by Jonsson et al. [17]) did not always capture the amount of time that predator and prey were in the same location, as assumed to translate into their likely frequency of interaction. We have, therefore, modified the microhabitat overlap index to account for this. Below, the entire, updated model is presented and described.

To account for differences in microhabitat use across species, we divide the mesocosm into microhabitat zones, quantify the amount of predation that occurs in each microhabitat zone, and sum across all zones. The amount of predation increases with the proportion of time spent in the microhabitat *m* (*p*_*i*, *m*_). When *p*_*i*, *m*_ is large, species *i* spends more time in microhabitat *m*, and if *p*_*i*, *m*_ and *p*_*j*, *m*_ are both large, we expect species *i* and *j* to encounter one another more often in microhabitat *m*. The encounter rate will also be affected by the size of microhabitat *m*; a fixed number of individuals in a larger microhabitat means a lower density and we therefore expect fewer encounters. To account for this, we divide *p*_*i*,m_*N_i_*p_*j*,m_*N_*j*_ by the proportional area (*A*_*m*_) of microhabitat *m* to get the adjusted encounter rate in microhabitat *m*. Here, we measure *p*_*i*, *m*_ empirically as the proportion of individuals of species *i* observed in microhabitat *m* (i.e. *p*_*i*,m_*N_*i*_), divided by the total number of individuals in the cage (*N*_*i*_).

We also introduce a term that describes the decrease in predation by a predator due to non-consumptive effects of other predators. This may include fear of predation, leading to decreased foraging, or physical interference [26, 28]. We propose that the magnitude of this effect depends on the likelihood of predator *j* being intraguild prey to predator *l*, and therefore depends on the expected attack rate of *l* on *j* (*a*_*jl*_). Microhabitat overlap will also affect predator encounters and should therefore affect the magnitude of non-consumptive effects (e.g. [22, 24]). We account for the effects of microhabitat overlap on non-consumptive predator-predator effects in the same way as described above for predator-prey interactions. In the functional response for predator *j* in microhabitat *m*, we sum over the potential attack rates of all species *l* on a single individual of species *j* to account for time spent avoiding or evading species *l* while species *j* is attempting to capture its own prey. The importance of non-consumptive predator-predator effects is described by the scaling constant *t*_0_, where a large value of *t*_0_ indicates a high decrease in successful attacks due to the time spent dealing with or avoiding non-consumptive effects decreasing the time available for searching or attacking. Non-consumptive effects from a conspecific individual may not be distinguishable from non-consumptive effects from another predator species, and so we remove the intraspecific competition term as used in Schneider et al. [3] from this version of the ATN model and replace it by the more general expression for non-consumptive effects from other predator individuals of any species.

In total, dynamics for the number of individuals *N*_*i*_ of species *i* are therefore given by:

$$\begin{array}{c} \begin{array}{cc} \frac{dN_{i}}{dt} & =r_{i}N_{i}-\sum_{j}\sum_{m}\frac{\frac{a_{ij}}{A_{m}}p_{i,m}N_{i}p_{j,m}N_{j}}{1+\sum_{k}\frac{a_{kj}}{A_{m}}h_{kj}p_{k,m}N_{k}+t_{0}\sum_{l}\frac{a_{jl}}{A_{m}}p_{l,m}N_{l}} \end{array} \end{array}$$ (1)

where species *i* increases in proportion to its intrinsic growth rate *r*_*i*_ (day^−1^) and decreases due to predation. We assume the intrinsic growth rate (*r*_*i*_) for predators to be zero due to their much longer generation time (a year) compared with the duration of our experiment (eight days). The realized per capita attack rate of predator *j* on species *i* in microhabitat *m* (*a*_*ij*_/*A*_*m*_) increases with the intrinsic attack rate determined by the predator-prey body-mass ratio (*a*_*ij*_, see below) and decreases with the size of the microhabitat, (*A*_*m*_), because predator and prey encounter each other less frequently in the larger area. Total predation in a microhabitat increases as the proportion of prey species *i* (*p*_*i*, *m*_ *N*_*i*_) and predator species *j* (*p*_*j*, *m*_ *N*_*j*_), in microhabitat *m* increases, but decreases dependent on the time predator *j* spends handling prey of the same or other species (*h*_*kj*_), or spends avoiding or interfering with other predators *l*.

As in Schneider et al. [3], we assume that for species body masses *W*_*i*_ and *W*_*j*_ (mg, corresponding to prey *i* and predator *j*), the allometric parameters (i.e. those dependent on body mass) are given by:

$$\begin{array}{c} \begin{array}{cc} a_{ij} & =a_{0}W_{i}^{1/4}W_{j}^{1/4}{\left(\frac{W_{j}/W_{i}}{R_{opt,j}}e^{1-\frac{W_{j}/W_{i}}{R_{opt,j}}}\right)}^{\phi }\\ h_{ij} & =h_{0}W_{i}^{1/4}W_{j}^{-1/4} \end{array} \end{array}$$ (2)

Allometric parameters assume that metabolism and movement speed scale with body mass [12]. While there is some variation [36], quarter-power scaling, as we use here, is pervasive across many biological rates and species [37]. This has allowed many models to utilize a simple approximation (3/4 rule) of the power to avoid estimating additional parameters. The derivations of these functions are described in greater detail by Schneider et al. [3]. We note the importance of scaling parameters *a*_0_, *h*_0_, and *R*_*opt*, *j*_. *a*_0_ scales the frequency of attacks when species encounter one another, with larger values of *a*_0_ indicating more frequent attacks. *h*_0_ scales the time spent handling alternative prey items. Handling time results in attacks on an increasingly low portion of the prey population as prey become more abundant; larger values of *h*_0_ indicate more time spent handling prey, which results in greater penalties to consumption rates. *R*_*opt*, *j*_ indicates the optimal predator-prey body-mass ratio for a successful attack by predator *j*, where *R*_*opt*, *j*_ = 1 indicates that predator *j* is most successful when attacking prey as large as itself and *R*_*opt*, *j*_ ≫ 1 or *R*_*opt*, *j*_ ≪ 1 indicates that predator *j* is most successful when attacking prey much smaller or larger than itself respectively. Parameter *ϕ* (*ϕ* > = 0) tunes the width of this success curve, indicating the importance of body size in determining feeding preferences. When *ϕ* = 0, attack rates are independent of prey size, while large values of *ϕ* mean feeding rates are highest close to *R*_*opt*_ and decrease away from this value. In contrast to Schneider et al. [3] and Jonsson et al. [17], we allow the value of *R*_*opt*_ to vary from predator to predator. This is to account for differences in traits not accounted for in the model that may affect predator foraging behavior. See S2 Table in S1 File for a summary of units for all parameters.

To determine the importance of the terms we introduce—microhabitat overlap and non-trophic predator-predator effects—we compare four variations of the model:

1. the full model (Eq 1, i.e. with *t*_0_ > 0 and including *p*_*i*, *m*_ and *A*_*m*_)
2. an intermediate model with microhabitat use but without predator interference (setting *t*_0_ = 0)
3. an intermediate model with predator interference but without microhabitat use (removing *p*_*i*, *m*_ and *A*_*m*_)
4. a minimal model without microhabitat use or predator interference (setting *p*_*i*, *m*_ and *A*_*m*_ to 1 and setting *t*_0_ = 0)

We fit each model to the data separately and so obtained different values for the fitted parameters for each model. The different parameters values affect (i) which prey a predator is most likely to consume and (ii) to what extent. As such, the values that we estimate here for *a*_0_, *h*_0_, *R*_*opt*_ etc. for our four model variants will, to some extent, reflect how each model emphasizes the importance of different factors for each interaction. *a*_0_ and *h*_0_ take the same value for all predators (within a given model), while the realized attack rate *a*_*ij*_ and handling time *h*_*ij*_ for each predator-prey pair is determined by body size of predator *j* and prey *i* (Eq 2). In contrast to *a*_0_ and *h*_0_, we allow *R*_*opt*_ to vary from predator to predator. A predator with an *R*_*opt*_ value of 1 will interact most strongly with prey of its own size, while a predator with an *R*_*opt*_ of 100 will more effectively consume prey 100 times smaller than itself. Next, if a predator spends all its time in a particular microhabitat, such as (in our case) on bean plants (*p*_*i*, *beans*_ = 1) it will have the strongest interaction with prey that also reside predominantly on beans, and have no interaction with prey that are never on beans. Crucially, when the model includes microhabitat use, it is the combination of both *R*_*opt*_ and *p*_*i*, *m*_ that dictates trophic interaction strength. For example, consider prey *x* (size = 10 and *p*_*x*, *beans*_ = 1) and prey *y* (size = 1 and *p*_*y*, *barley*_ = 1) with predator *i*. If predator *i* (size = 100 and *p*_*i*, *beans*_ = 1) only interacts with prey *x*, this is already captured entirely by the microhabitat use terms since the predator spends all its time in the same microhabitat zone as prey *x* and never overlaps with prey *y*. In a model accounting for microhabitat use (as in models 1 and 2), *R*_*opt*, *i*_, therefore, will likely have an estimated value near 10, since predator *i* is ten times larger than prey *x*. If, however, we do not account for microhabitat use (as in models 3 and 4), the model needs some other way to capture that predator *i* does not interact with prey *y* in order to optimize the fit to empirical data. In the parameter estimation this could be achieved by a larger value of *R*_*opt*, *i*_ (moving its optimal prey size further from prey *y*), or a higher value of *ϕ* (narrowing the effective feeding range), thus absorbing differences based on which terms are present in the model and producing good model fit without necessarily reflecting the ‘true value’ of a parameter and thus realism in the interaction between predator *i* and prey *y*. To guard against this, we impose boundaries on the values parameters can take, preventing the model from taking on unrealistic parameter values in such cases.

We do not explicitly include non-consumptive mortality in this model. For the aphid (or basal) prey, mortality not due to predation is included in the growth rate term *r*_*i*_, while for predators the experiment is not long enough that we expect mortality other than that due to intraguild predation. Furthermore, without single individual controls, it would be difficult to separate “natural” mortality from that due to predation or cannibalism.

## Methods

### The mesocosm experiment

**Fig 1:**
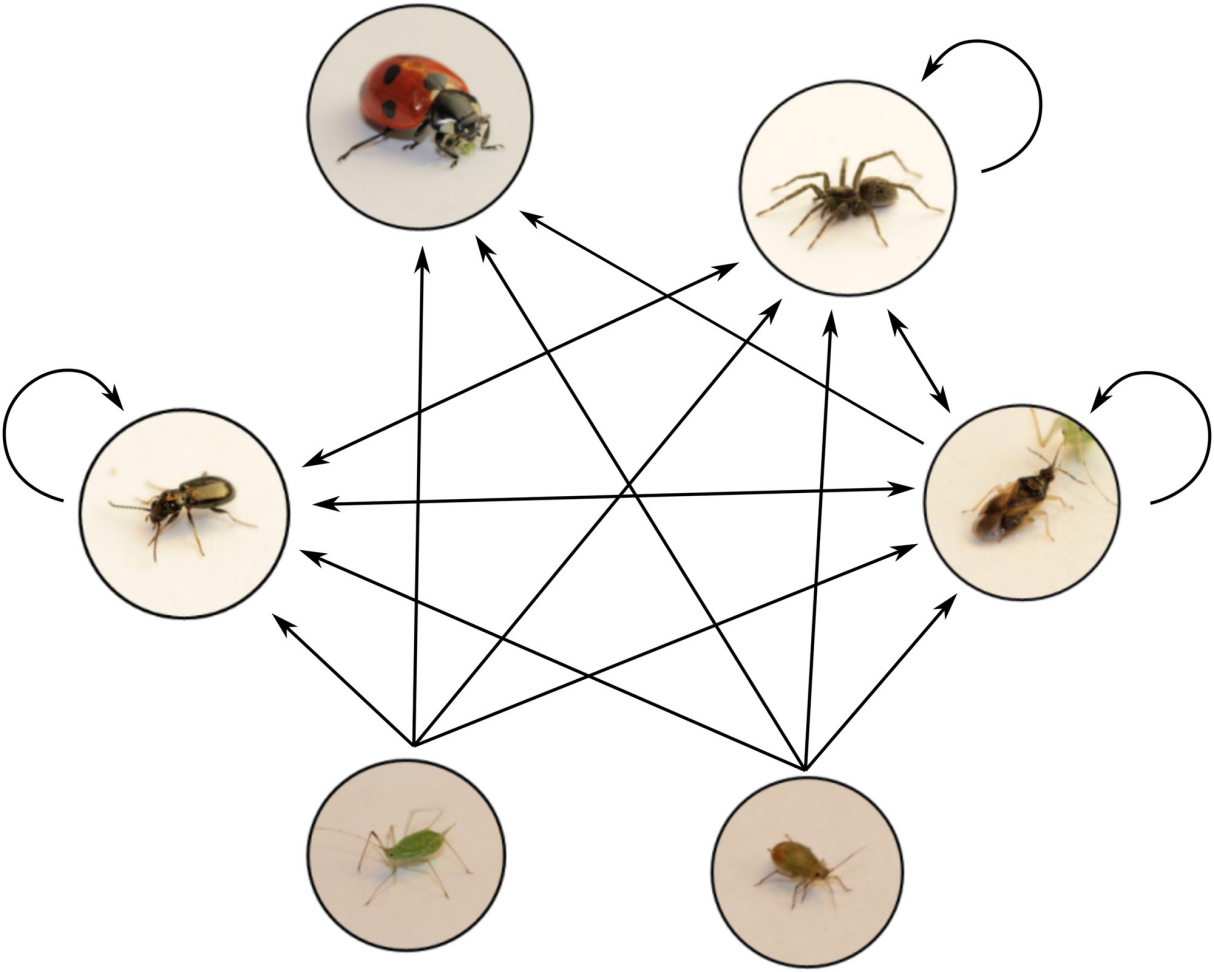
The food web including all possible interactions that we allowed in the model. Species are, from top left: lady beetle (*Coccinella septempunctata*); wolf spiders (*Pardosa* spp.); minute pirate bug (*Orius majusculus*); bird cherry-oat aphid (*Rhopalosiphum padi*); pea aphid (*Acyrthosiphon pisum*); and ground beetle (*Bembidion spp*). Arrows indicate potential feeding interactions which we then parameterize through least squares minimization (Section 3.2). Arrows point from prey to predator. Double headed arrows indicate that species could potentially eat each other and arrows beginning and ending with the same species indicate cannibalism. We removed all interactions to and from *C. septempunctata* except for *C. septempunctata* preying on aphids and *O. majusculus*, and assumed that the aphids did not consume any predators. This arthropod community was dependent on two species of plants; barley (*Hordeum vulgare*) and fava beans (*Vicia faba*).

To empirically test and parameterize this model required a study system with rapid growth of the prey population, a range of body sizes of both predators and prey, and distinct microhabitat zones. With this in mind, and with the benefit of data from a previous experiment [17], we assembled a six-species terrestrial arthropod community [30] (Fig 1) dependent on two species of plants; barley (*Hordeum vulgare*) and fava beans (*Vicia faba*).

**Fig 2:**
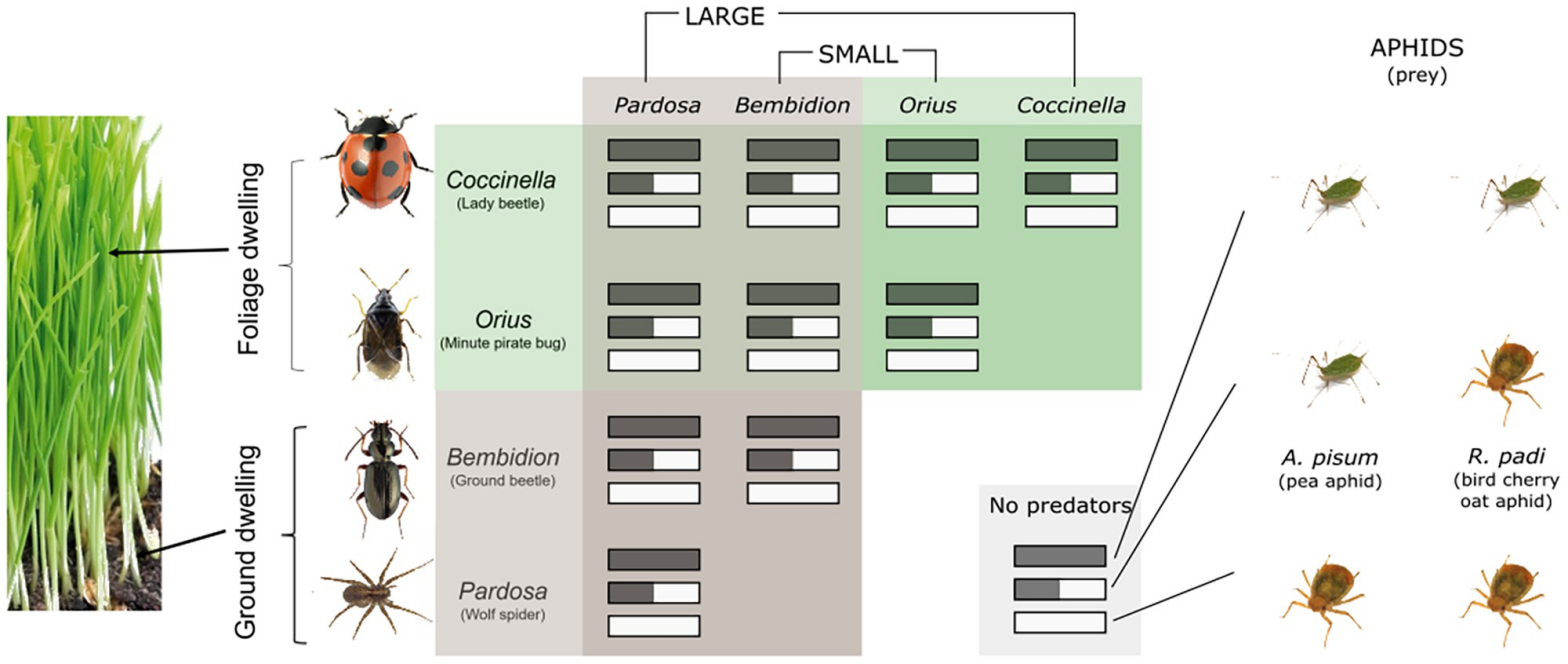
An overview of the predator-prey combinations used in the experiment. Each combination was replicated six times. Figure replicated from [30].

As primary consumers we chose one large (*Acyrthosiphon pisum*, 0.67mg) and one small (*Rhopalosiphum padi*, 0.155mg) species, both aphids. Next, to explore the importance of body mass and microhabitat use in trophic interactions, we chose four predators on these prey, differing in body size and/or microhabitat preference; one large and one small predominantly foliage-dwelling predator (*Coccinella septempunctata*, 37mg, and *Orius majusculus*, 0.58mg), and one large and one small predominantly ground dwelling predator (*Pardosa* spp., 18mg, and *Bembidion* spp., 2.15mg, where spp. signals the potential inclusion of several congeneric but morphologically indistinguishable species). Each mesocosm contained both barley (as a host for *R. padi*) and fava beans (as a host for *A. pisum*), one or both aphid species, and zero, one or two predator species. All combinations of predator and prey were replicated six times in a fully factorial design (Fig 2). This resulted in 30 predator-prey combinations, plus three control treatments with no predators. This resulted in a total of 198 mesocosms, which were run in four batches. Treatments were spread such that each treatment occurred at least once and no more than twice in each batch. Each run lasted 8 days from when predators were added, and mesocosms were replanted before each run.

Plants were sown in 60x40cm, 20cm deep, plastic containers, each with two rows of 10 fava bean seedlings (20 in total per container) and three rows of 15 barley seedlings (45 in total). A 60cm high mesh cage, with one side resealable to allow aphid counting, was placed on top of each container to prevent insects from entering or escaping the microcosm.

150 wingless adult aphids, taken from a colony maintained in the lab, were introduced per microcosm on Petri dishes two days before the experiment began. One third of the mesocosms (66 mesocosms) were inoculated with 150 *R. padi* (zero *A. pisum*), one third with 150 *A.pisum* (zero *R. padi*), and the final third with 75 *R. padi* and 75 *A. pisum*. Predators were introduced at the beginning of the experiment. The number of predators was determined using a combination of short term (8hr) feeding trials and pilot studies to reach a density where predators would impact the prey, but not eliminate them too quickly. Predator numbers in single-species mesocosms were: *C. septempunctata*: 4 individuals; *O. majusculus*: 40 individuals; *Pardosa*: 20 individuals; *Bembidion*: 40 individuals. Mesocosms with two predators species contained half the number of individuals of each predator species as single predator-species mesocosms, i.e. mesocosms with both *Pardosa* and *Bembidion* together contained 10 *Pardosa* individuals and 20 *Bembidion* individuals. This substitutive design was to avoid the effect of doubling the density of predators. *O. majusculus* were ordered from Lindesro AB, while *C. septempunctata*, *Pardosa* and *Bembidion* individuals were collected from fields surrounding Uppsala, Sweden.

Frequency and timing of aphid counts were determined based on our pre-experimental analyses [30], but by using in-cage rather than destructive sampling, we were able to increase sampling slightly from the minimum determined in Laubmeier et al. [30]. Aphid populations were counted on days 2, 4, 6 and 8. Treatments with *C. septempunctata* were also counted on days 1 and 3, because we realized that *C. septempunctata* decimated aphid populations so rapidly that we would require more data points in order to obtain an estimate of their parameters. Aphids were counted by opening the cage door and carefully counting the number of aphids on each plant.

**Fig 3:**
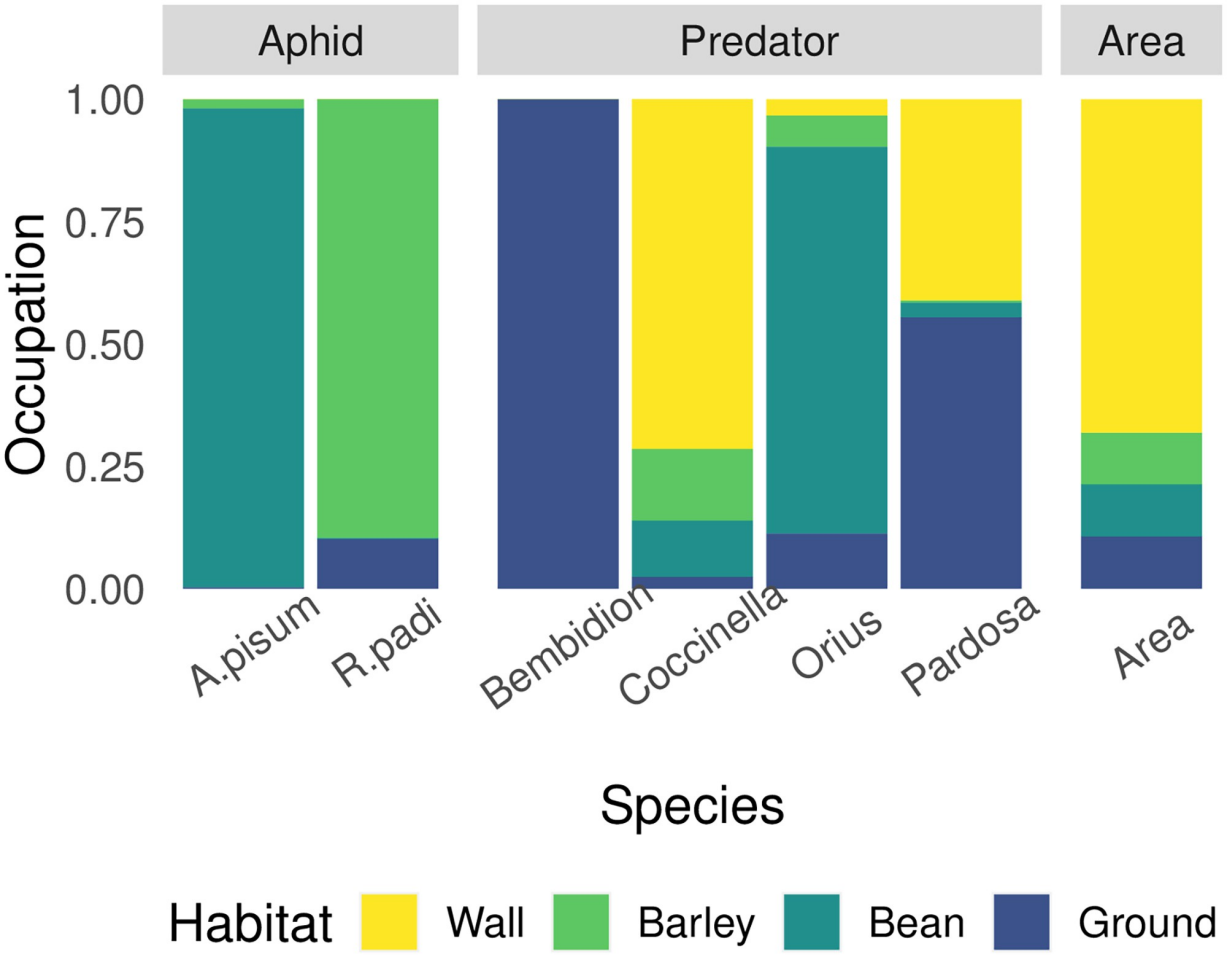
Microhabitat preferences as estimated from the proportion of time each species spent in each of the four microhabitat zones. The final column shows the relative size of each area in the experimental cages.

The proportion of time predators spent in each microhabitat, *p*_*j*, *m*_, was measured in single predator mesocosms. While it is possible that predators will change their microhabitat use in the presence of other predators, our intention here is to model interactions with relatively few parameters, and in particular to make it possible to extend to new species without having to measure parameters for each new species’ combination. We expect that predators will be most likely to change their microhabitat use in response to the potential for intraguild predation, the effect of which should therefore be absorbed by the parameter for non-trophic predator-predator effects (*t*_0_). When measuring microhabitat use, the location of each predator was marked on a mesocosm map before beginning to count aphids in these mesocosms. In these counts, between 20% and 100% of initial predators were found. In 3/4 of cases over 50% were found. We then categorized these into four areas: walls/roof, ground, beans, and barley. Aphid microhabitat use was measured by separating aphid counts into each of those categories, but only recorded on days 2 and 6. While the absolute area of beans and barley changed throughout the experiment, we estimated that, on average, the surface area of beans, of barley, and of the ground were roughly equivalent, while the combined area of the walls and roof was six times larger than each other microhabitat area (Fig 3).

Predators could only be reliably counted through destructive sampling of the mesocosms, and were therefore only counted on the final day (day 8). After the aphid count, predators were collected by a thorough examination of cage and plants, and sifting through the soil. An additional predator search was repeated the next day to catch any missed in the initial search.

Over the duration of the experiment, we found that *C. septempunctata* could occasionally escape through gaps in the mesh cages. We assume that any *C. septempunctata* missing from cages escaped in this manner, as other predators were never observed consuming *C. septempunctata*. *C. septempunctata* were more easily observed than other predators, so we could be certain that we had counted all individuals present in the cage. Because this change in the population is not described by our mathematical model, we added replacement individuals to cages where *C. septempunctata* went missing and did not dynamically model the population. Instead, we directly input *C. septempunctata* population densities into the model for other species’ population dynamics. We fixed these densities at constant levels for the duration of the experiment by taking the average value of all observed abundances in each cage. We used averages instead of time-series data due to the uncertainty associated with our observations; it was impossible to know exactly when between observations the individuals went missing from the mesocosm.

The ATN model describes species interaction strengths as a function of species traits (in our case body size and microhabitat use). Because the presence or absence of a food-web link is simply a binary interpretation of interaction strength, the ATN model also predicts the binary food-web structure. However, if a feeding interaction is prohibited due to traits not accounted for in the model, it cannot be expected to correctly predict the absence of such links. As neither body size nor microhabitat could explain why the other predators did not consume *C. septempunctata* (there was microhabitat overlap and predation of similar-sized intraguild prey), we removed feeding interactions on *C. septempunctata* from other predators from the network of potential interactions (Fig 1). Similarly, *C. septempunctata* did not consume *Bembidion* or *Pardosa*, for reasons not necessarily explained by microhabitat use or body size (most likely *Bembidion*’s hard cuticle (e.g. [18]) and *Pardosa*’s speed), so we removed these interactions.

### Model fitting

Using abundance data from our experiment, we parameterized four versions of the ATN model (with versus without microhabitat use and predator interference, see Section 2 The model). We use least squares minimization to search for parameters that minimize a “least squares” cost, which describes the deviation of model predictions from empirical observations [38]. This is a common and well-established approach to parameterizing biological models [39–41].

Although this method of parameterization permits weighted statistical models for error in the data [38], we were unable to determine a meaningful weighting from the available data. We therefore rely on an ordinary least squares cost, in which we assume that observational error and process noise is normally distributed with unknown variance. Under this approach sparse or noisy data can give rise to multiple or unreliable estimates [42, 43]. To guard against this possibility, in [30] we *a priori* explored population dynamics to determine optimal and minimal timing and frequency of experimental sampling. Population sensitivity to model parameters impacts parameter estimates and is often used to assess identifiability and certainty *after* model fitting (for example, through use of the Fischer Information Matrix in [44] or [45]). We therefore selected a sampling strategy to maximize model sensitivity (reducing the potential for non-identifiable or uncertain parameters) and arrived at an optimized design for generating empirical data to inform our candidate models [30].

We minimized the least squares cost across all possible parameterizations, using the numerical optimization function fmincon and ODE solver ode45 in matlab version 9.8.0.1417392 (R2020a). We constrained the parameter space for the minimizing search according to the range of observed parameters available in the literature (see Supplemental Methods, S1 Table in S1 File) and utilized the multistart function to guard against local minimization, by numerically solving the minimization problem from many different starting points in the available parameter space. In order to compare the importance of microhabitat use (*p*_*i*, *m*_) and non-trophic predator-predator effects (*t*_0_), we repeated this fitting for each of the four models (with versus without *p*_*i*, *m*_ and *t*_0_).

To fit the models, we first established a common baseline for aphid growth. Using the data from predator-free control treatments, we estimated initial aphid abundances and the intrinsic growth rate (*r*_*i*_) for *A. pisum* and *R. padi*. The intrinsic growth rate in the absence of predators differed between the two aphid species, but we assumed that each aphid species’ growth rate did not change across all aphid treatments (single-species or combined) and replicates. We assumed a different initial abundance for each aphid species and aphid treatment (i.e. *R. padi* alone versus in combination with *A. pisum*, and *A. pisum* alone versus in combination), but used the same initial abundance across all replicates of the same aphid treatment type. Because predators were added the day the experiment began (rather than two days earlier as aphids were), we used the known predator abundances rather than estimating them. After estimating aphid growth rates and initial abundances from predator-free mesocosms, we estimated the remaining model parameters using data from predator-treated mesocosms. There were 10 predator treatments: each of the four predator species alone, plus each pairwise combination. Each predator treatment was replicated with 18 aphid populations; 6 replicates each of *A. pisum* and *R. padi* in isolation, as well as 6 replicates of *A. pisum* and *R. padi* in combination with each other (Fig 2). We simultaneously estimated constants for allometric relationships (*a*_0_, *h*_0_, *R*_*opt*, *j*_, *ϕ*) and predator interference (*t*_0_) using all predator-treated data in aggregate, while keeping aphid initial abundances and intrinsic growth rates (*r*_*i*_) fixed at previously estimated values. Model estimation used a constrained optimization algorithm, described in detail in the Supplementary Material. For the common values between all models (*r*_*i*_ and initial abundances), we placed bounds on the values that initial abundances could take using observed initial abundances in the experiment and informed the bounds for *r*_*i*_ from prior estimates of aphid intrinsic growth rates. For each separate model, we placed bounds on the values that the parameters could take based on values reported in the literature when possible (for *h*_0_ and *R*_*opt*, *j*_), and repeated the estimation over multiple bounds to ensure a true minimization for remaining parameters (for *a*_0_, *t*_0_, and *ϕ*) (see S1 Table in S1 File).

A fundamental goal of trait-based modelling is to yield flexible parameterizations for predator-prey interactions with fewer parameters to estimate. Scaling constants must therefore be the same across all treatments and replicates, and variability across treatments must emerge from differences in predator traits (and *R*_*opt*_ values which account for traits not explicitly included in the model). By using the same parameterization and initial abundance across treatments and replicates, and restricting the values that parameters can take, we limit the flexibility of the model to fit to the data. We will not capture differences in population outcomes due to external, potentially stochastic factors, such as variation in plant growth. Although the resulting fit to data may be worse, it allows us to discern which models, and therefore which parameters, best explain the data. For the reduced models, we utilized the same values for *r*_*i*_ and initial abundances as in the full model and repeated the process for estimating remaining parameters. To remove predator interference, we set *t*_0_ = 0 and did not estimate that parameter. To remove microhabitat use, we set *p*_*i*, *m*_ = 1 and *A*_*m*_ = 1, removing the summation over all *m*.

### Model evaluation and prediction

The above steps utilize the entire data set to derive parameter estimates. Each estimation problem yields a cost criterion (JLS) quantifying model fit, where a lower value of JLS indicates a better model fit. To calculate relative model performance (i.e. accounting for models which have more parameters), we also calculate the Akaike information criterion (AIC). We can further evaluate the performance of each model according to the realism of estimated parameter values and associated processes (e.g. feeding rates), compared to literature or supplemental empirical testing. To summarize, we used a mix of statistical and expert-knowledge model performance criteria, specifically: (i) metrics of model fit (the JLS cost criterion and AIC), (ii) visual fit of predicted vs observed aphid abundance, (iii) realism of parameter values, (iv) realism of ecological processes, and (v) observed vs predicted final predator abundance.

A fundamental aim of trait-based dynamic modeling is to predict new species or scenarios. To demonstrate how the models might compare in their predictions for a new prey species, we used each model, with its resulting parameterization, to predict the population on days 2, 4, 6, and 8 of a hypothetical, entirely ground-dwelling prey species weighing 1mg (slightly larger than *A. pisum*) paired with each of our four predator species. Allometric parameters (*a*_*ij*_, *h*_*ij*_) for the interaction between this new hypothetical prey species and each predator under the different model alternatives were obtained by applying the fitted values of scaling parameters (*R*_*opt*_, *a*_0_, *h*_0_) in Table 1 to Eq 1 (together with the ‘actual’ body size of the predator in question and body size of the new hypothetical prey). This exercise demonstrates that i) the difference between models may become most apparent outside the range of data they are fitted to, and ii) that this is particularly important if we wish to use our models to predict new species’ dynamics. Applying this approach to real new species would be one way to test which model is most accurate. Nonetheless, it would call for a massive investment in terms of working hours, and is therefore outside of the scope of the current study.

**Table 1:** Parameter values (±95% confidence intervals) and model fit (JLS and AIC) for models with and without microhabitat use and non-consumptive predator-predator effects.

Model	1	2	3	4
*r*_*R*.*padi*_	0.3787 ± 0.135			
*r*_*A*.*pisum*_	0.3454 ± 0.078			
*a*_0_	9.56 ± 4.25	1.47 ± 0.48	25* ± 10.95	25* ± 25.68
*h*_0_	0.03 ± 0.01	0.01 ± 0.01	0.02 ± 0.00	0.42 ± 0.43
*t*_0_	10.8 ± 4.63	-	13.1 ± 5.72	-
*ϕ*	1.34 ± 0.01	1.35 ± 0.48	0.35 ± 0.00	0.05 ± 0.00
*R*_*opt*_, *P*	50.4 ± 2.2	34.0 ± 3.4	100* ± 0.03	100* ± 1.7
*R*_*opt*_, *O*	6.36 ± 0.19	106 ± 142	18.8 ± 0.1	175* ± 8
*R*_*opt*_, *C*	250* ± 6.3	250* ± 63	250* ± 1	250* ± 4
*R*_*opt*_, *B*	200* ± 9.2	200* ± 122	190.6 ± 0.8	200* ± 7
JLS	7.41e+08	7.71e+08	8.23e+08	1.02e+09
AIC	13,857	13,896	13,965	14,182

* Estimate at the bounds of the constrained minimization. See S2 Table in S1 File.

Model 1 = full model with both microhabitat and non-consumptive effects. Model 2 = only microhabitat. Model 3 = Only non-consumptive effects. Model 4 = minimal model, neither microhabitat nor non-consumptive effects. *r*_*R*.*padi*_ and *r*_*A*.*pisum*_ refer to the intrinsic growth rates of the aphids *R. padi* and *A.pisum* respectively and are estimated once and then have the same value across all models. *a*_0_, *h*_0_, *t*_0_ and *ϕ* refer to parameters in Eqs 1 and 2. *R*_*opt*_, *P*, *R*_*opt*_, *O*, *R*_*opt*_, *C* and *R*_*opt*_, *B* refer to the optimal predator-prey body-size ratio for *Pardosa* spp., *O. masculus*, *C. septempunctata*, and *Bembidion* spp. respectively.

## Results

Based on an integration of our assessment criteria of (i) metrics of model fit (the JLS cost criterion and AIC), (ii) visual fit of predicted vs observed aphid abundance, (iii) realism of parameter values, (iv) realism of ecological processes, and (v) observed vs predicted final predator abundance, the full model (model 1) performed the best, followed by the model with microhabitat but not non-trophic predator effects (model 2), then the model with non-trophic predator effects but not microhabitat (model 3), and finally the model with neither non-trophic predator effects nor microhabitat (model 4). Here we explain each criterion in turn and how the models perform under them.

Based on the **metrics of model fit** (Table 1), the full model best explains the experimental data (JLS = 7.41E+08, AIC = 13,857.00), closely followed by the model with microhabitat but not non-trophic predator effects (JLS = 7.71E+08, AIC = 13,896.00). The model with only non-trophic predator effects is the third best performer, while the model with neither non-trophic predator effects nor microhabitat performed the worst. We see the largest confidence intervals for the parameters in the model with microhabitat but not non-trophic predator effects, especially for *R*_*opt*_ values (Table 1). This means that, while this model is one of the better performing models according to JLS and AIC, we are much less confident in some of its parameter estimates. The other models have confidence intervals roughly similar to each other. However, for parameters at the bounds of the constrained optimization, such as *R*_*opt*, *C*_, a narrow confidence interval does not suggest that the estimated values are correct. Parameters at the bounds of the optimization suggest a disconnect between the model and data, for example a missing trait or an incorrect assumption about prey preferences.

**Fig 4:**
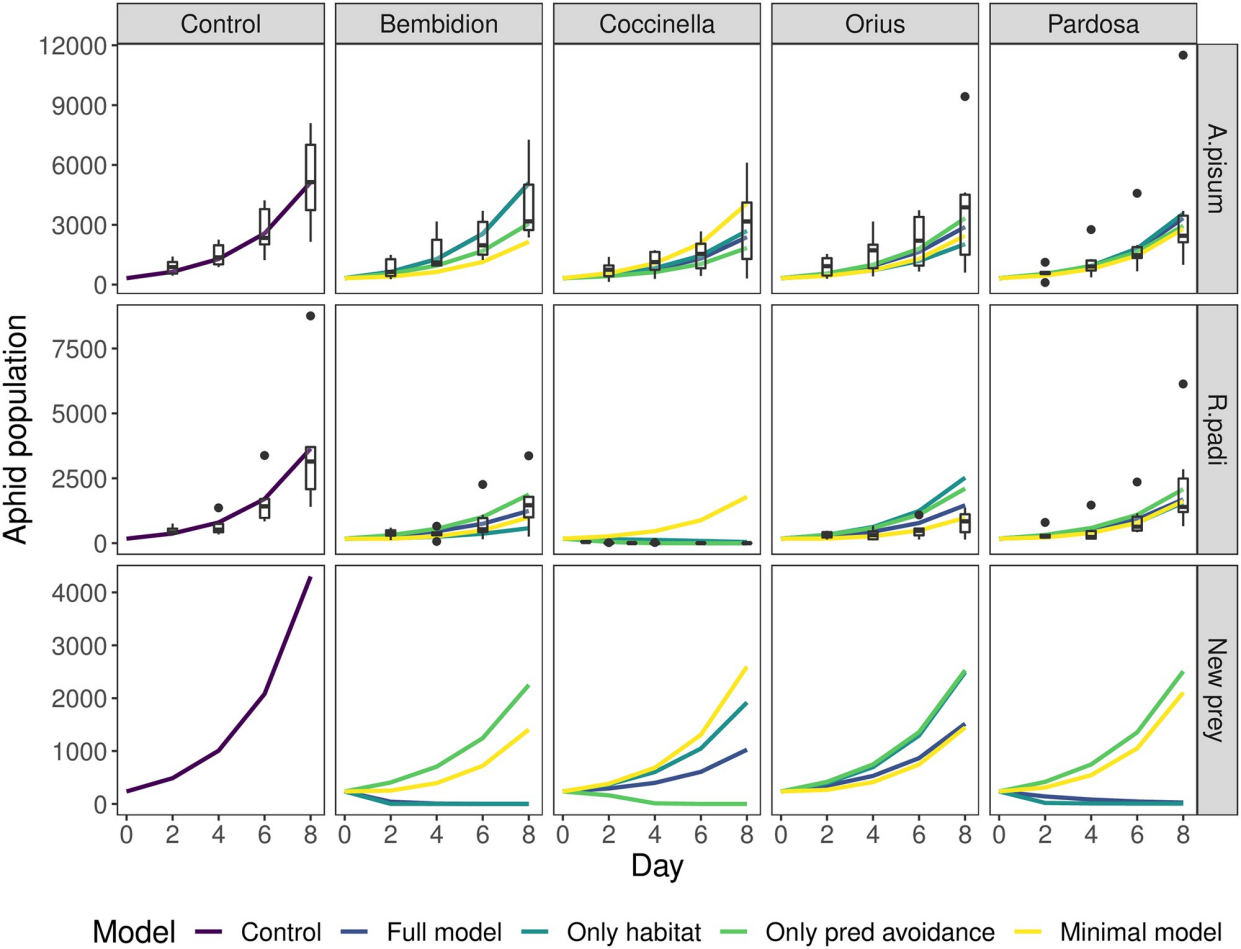
Model predictions of aphid population growth (lines) across time, compared to data of aphid counts per day (boxes) in single-aphid (rows) single-predator (columns) treatments. Boxes show first and third quartiles and outlying points indicate data further from the hinge than 1.5 times the interquartile range. Lines show predictions of the different models. The final row shows model predictions when including a hypothetical new prey species that resides entirely on the ground and has a body size of 1mg. The closer a model trajectory is to the mean of the experimental data (horizontal line within boxes) the better a particular model performs. A model underestimates (overestimates) the effect of a predator (columns) on an aphid prey (rows) when its trajectory value for the prey is greater (smaller) than the experimentally observed data.

Based on the **visual fit of predicted vs observed aphid abundance**, all models perform relatively well (Fig 4). The full model is usually the best and never an outlier in poor performance. In contrast, the minimal model is the only model to get the direction of population growth wrong (predicting an increase of *R. padi* with *C. septempunctata*), underestimates the impact of *C. septempunctata* on *A. pisum* and overestimates the effect of *Bembidion* on *A. pisum*. The worst performance for the model with only microhabitat and the model with only non-trophic predator effects is for *O. majusculus* on *R. padi* where they both underestimate the impact of *O. majusclulus*. For *Bembidion* with *R. padi*, the model with only non-trophic predator effects underestimates the effect of the predator while the model with only microhabitat overestimates the effect of the predator. This suggests that for the *O. majusculus-R.padi* and *Bembidion-R.padi* interactions in particular, both microhabitat and non-trophic predator effects are important for the interaction.

With respect to the **realism of parameter values**, we do restrain the values to fall within (generously set) realistic ranges. As a result, all estimated parameter values (Table 1) are in that respect ‘realistic’. However, in models without microhabitat use, many parameters hit the boundaries we set (indicated by * in Table 1). This suggests that if we did not set those boundaries, these parameters would have been driven towards unrealistic values for those models. This is particularly true of *R*_*opt*_ (the optimal predator-prey body-size ratio) parameter values, which hit, or nearly hit, their upper limit for most predators in models without microhabitat use. However, in these models, *ϕ* is also very low which means that predators have similar attack rates on prey of all sizes and body size is not important in driving interaction strengths. Predators can indeed have a very wide range of body sizes they can consume. But we know, at least for some predators, that body size does play an important role and that there are limits (e.g. [3, 17, 46]). So it is likely that *ϕ* is smaller than it realistically should be in these models. The combination of *R*_*opt*_ values hitting their boundaries and low *ϕ* suggests that, without microhabitat in the model, the model struggles to use body size to explain dynamics, at the expense of parameter realism. With respect to particular *R*_*opt*_ values, we know from observation that both *Pardosa* and *Orius* are cannibalistic and oftentimes consume prey or conspecifics of a size similar to themselves. This means we would expect small *R*_*opt*_ values. In models with microhabitat use, *Pardosa* does have relatively low *R*_*opt*_ values (50 and 34, meaning that *Pardosa* will preferentially eat *A. pisum* (ratio = 26) and *O. majusculus* (ratio = 30)), while *Orius* has the lowest *R*_*opt*_ values in models with non-trophic predator effects (6.36 and 18.8; the body-size ratio for *O. majusculus* with *R. padi* is 4) and a higher *R*_*opt*_ value in the model with only habitat use (106). In the other models, *R*_*opt*_ for both predators hits, or nearly hits, its upper bound.

**Fig 5:**
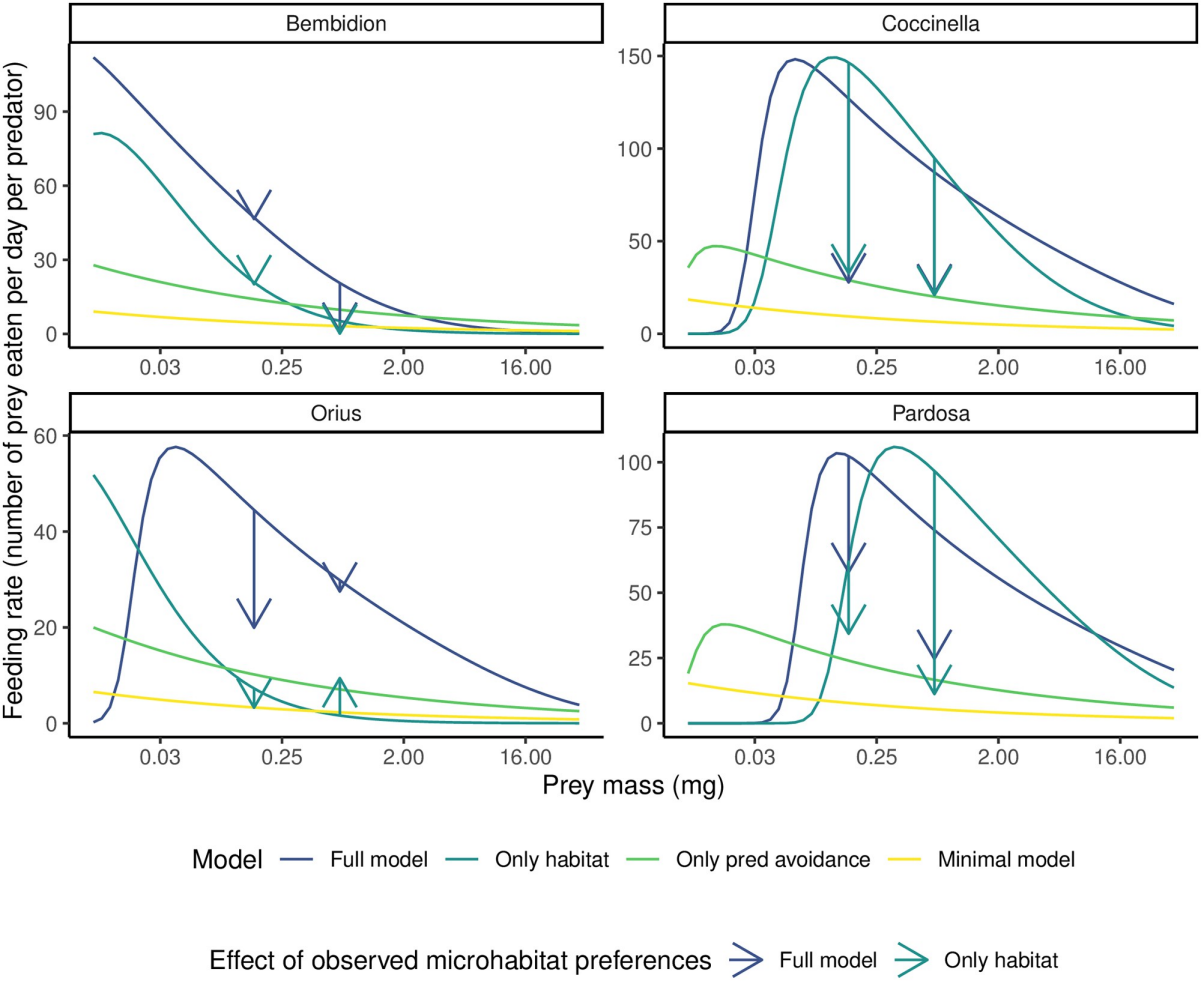
Model predictions for an individual predator’s feeding rate (number of prey consumed per predator per day, y axis) on prey of different body sizes (x axis). Curves show the “microhabitat-preference free” feeding rate, i.e. assuming all species use all microhabitats in proportion to their area. Vertical arrows show the difference in feeding rate when accounting for observed microhabitat preferences of the predator with aphid prey (*R.padi*, mass = 0.155mg and *A.pisum*, (mass = 0.67mg). The arrow tip shows the predicted feeding rate of the predator on a prey of that size *when accounting for microhabitat preferences*. A longer vertical arrow therefore means that observed microhabitat preferences have a larger effect on feeding rate. Line color corresponds to different models. Models with microhabitat use (blue and dark green lines) predict the highest “microhabitat-preference free” feeding rates, but when actual microhabitat preferences are accounted for their feeding rates drop closer to that predicted by the other models. We show the instantaneous feeding rate with a population of 250 aphids and in the *absence* of any other predator individuals (i.e. not accounting for non-trophic predator effects. To see their effect, compare with S1 Fig). Observe that, for *Bembidion*, accounting for microhabitat use of *R.padi* does not substantially change the predictions of models with microhabitat (i.e. the arrows sit on the curve). This is because the overlap of *Bembidion* with *R.padi* works out to be almost the same as if they used all microhabitats in proportion to the size of the microhabitat. Note the varying scales of the y-axis.

In terms of the **realism of ecological processes**, we can look at feeding rates (Fig 5) and handling times (Table 2). Overall, models with microhabitat use show the highest ‘microhabitat-preference free’ feeding rates on aphids (i.e. assuming each species uses each microhabitat in proportion to its area rather than taking account of any microhabitat preferences of either prey and predator, shown by the blue and dark green curves in Fig 5). This is because differences in microhabitat use usually decrease the *realized* feeding rate relative to the ‘microhabitat-preference free’ rate (see arrows showing the change when microhabitat use is accounted for in Fig 5). Furthermore, intraguild effects among predators are important. We can see from Fig 5 that the full model predicts the highest realized feeding rates of all models on aphids (34–77 aphid individuals consumed per day per predator, depending on the predator) *when no other predator individuals are present*. If we account for the presence of other predators and the decrease in foraging caused by interference and the fear of predation, these rates can drop dramatically (to 2–34 aphids per predator per day, see S1 Fig). This drop in realized feeding rate is especially true of *Pardosa* and *O. majusculus*, both whom are cannibalistic.

**Table 2:** Handling times (*h*_*ij*_, units = ind_*i*_/day) for each predator with *R. padi* and *A. pisum* for each model.

Predator	Prey	Model 1 (h = 0.03)	Model 2 (h = 0.02)	Model 3 (h = 0.14)	Model 4 (h = 0.42)
*Bembidion*	*R. padi*	0.014	0.008	0.073	0.218
*C. septempunctata*		0.007	0.004	0.036	0.107
*O. majusculus*		0.019	0.011	0.101	0.302
*Pardosa*		0.008	0.005	0.043	0.128
*Bembidion*	*A.pisum*	0.020	0.011	0.105	0.314
*C. septempunctata*		0.010	0.006	0.051	0.154
*O. majusculus*		0.028	0.016	0.145	0.436
*Pardosa*		0.012	0.007	0.062	0.185

Model 1 = full model with both microhabitat and non-consumptive effects. Model 2 = only microhabitat. Model 3 = only non-consumptive effects. Model 4 = minimal model, neither microhabitat nor non-consumptive effects.

Importantly, the feeding rates in Fig 5 are the intrinsic rates of feeding when there is a fixed population of 250 aphids and no other predator individuals present. As the aphid and predator populations change, these feeding rates will change substantially (see S1 Fig to see how the feeding rate changes in response to other predators and the resulting non-trophic predator effects). This makes it somewhat difficult to pinpoint exactly how realistic the feeding rates are, but all models predict rates roughly within those known from the literature [47–50]. Model predicted handling times (Table 2) vary from 0.004 days (four and a half minutes) per prey per predator for *C. septempunctata* and *Pardosa* consuming *R. padi* in the model without fear of predation, to 0.44 days (11 hrs) per prey per predator for *O. majusculus* consuming *A. pisum*. Based on supplementary experiments during the experiment, we observed that one hour (0.04 days) is approximately the longest that any predator (*O. majusculus* in particular) takes to consume prey. This means that the handling times for the full model and the model without non-trophic predator effects are the most realistic (Table 2). The minimal model predicts very long handling times (0.11–0.44 days per prey per predator), which are inconsistent with direct observations of feeding behaviour in the mesocosms and indicate the minimal model is accounting for predator interference or habitat overlap through increased handling times.

**Fig 6:**
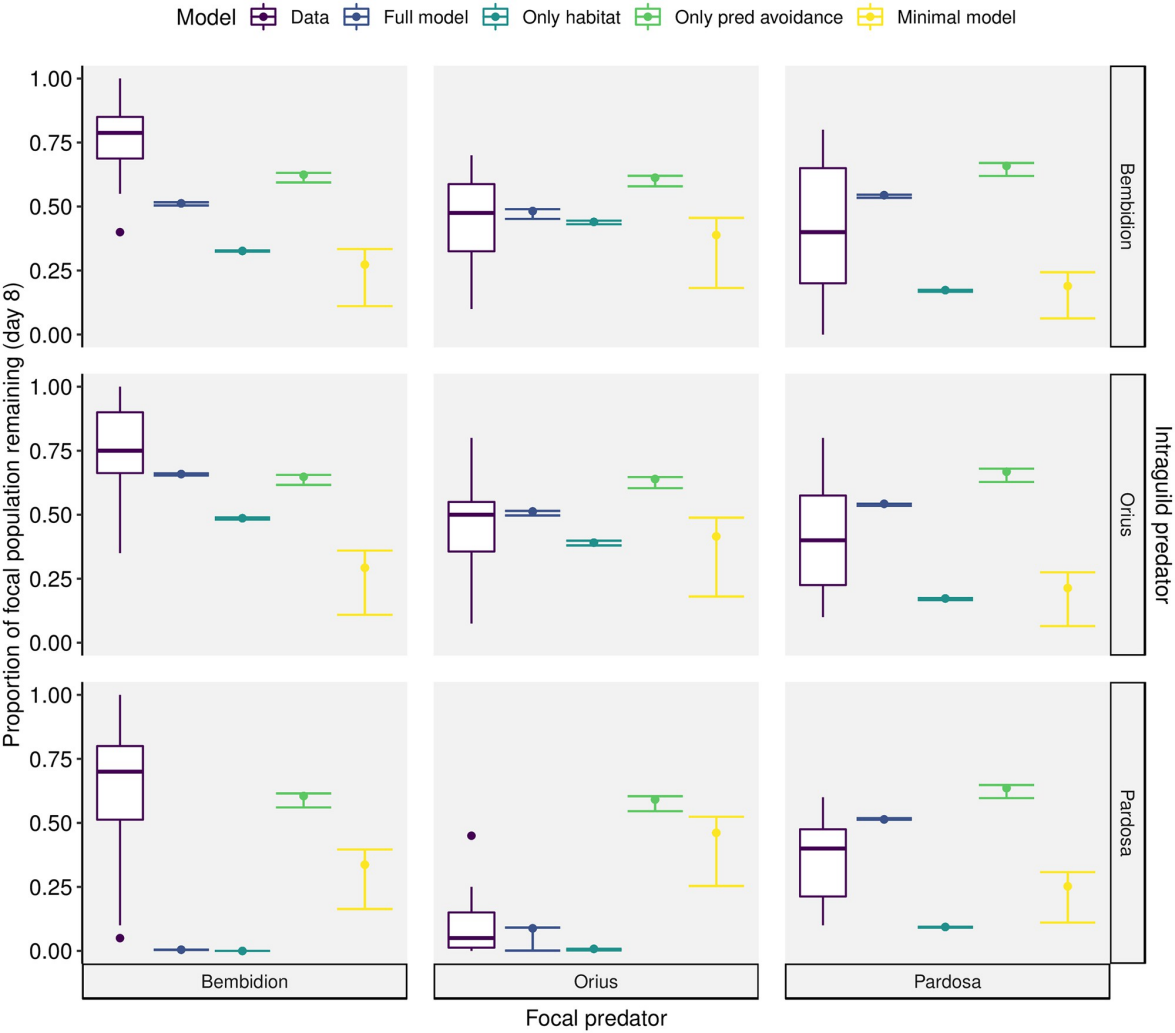
Experimental results (purple boxes) and model predictions for the proportion of each (focal) predator population surviving on the final day of the experiment when combined with different intraguild predators (i.e. the top right panel shows the proportion of the *Pardosa* population remaining at the end of the experiment when combined with *Bembidion*). The experimental data is shown as a box plot, showing the 25th, 50th, and 75th quartiles. Model predictions depend on the focal and intraguild predator, but also on the prey treatment. The three prey treatments are shown as a point and error bars; where all three are stacked, prey treatment makes no difference to the predicted predator population. Note that *C. septempuntata* was not modelled dynamically and did not predate on most other predators, so we exclude it from this plot.

When it comes to **observed vs predicted final predator abundances** (Fig 6), the full model is usually the best. The full model does, however, overestimate the effect of *Pardosa* on *Bembidion* (predicting no survivors, but actually ∼75% survived). The model with only habitat tends to overestimate the consumptive effect of predators on other predators (intraguild predation), predicting smaller final predator populations. This is especially true of final *Bembidion* and *Pardosa* populations. The model with only non-consumptive predator effects tends to underestimate the effect of intraguild predation, especially on *O. majusculus* and *Pardosa*. The minimal model also tends to overestimate the effect of intraguild predation, except on *O. majusculus*. This model also has the biggest variation in predictions depending on aphid treatment, which is due to the much higher handling times compared to the other models.

When it comes to making predictions **outside the range of our data**, we see that the models predict very different impacts of each predator on a hypothetical, entirely ground-dwelling prey species (i.e. very different from the foliage dwelling aphid prey) slightly larger than *A. pisum* (Fig 4). *Bembidion* and *Pardosa* are predominantly ground-dwelling and would share the same microhabitat as the hypothetical prey, so models which account for microhabitat predict that these predators will strongly drive down the population of this prey, while models which do not include microhabitat predict much weaker effects. *O. majusculus* is predicted to have the weakest effect where only non-trophic predator effects or microhabitat is accounted for (because they will be busy avoiding each other or have little microhabitat overlap), and *C. septempunctata* displays a range of effects.

## Discussion

In Laubmeier et al. [30], we developed a dynamic food-web model, taking into account body size, microhabitat use, and non-consumptive predator-predator effects, and then ran pre-experimental simulations to determine the optimal experimental design. Here, we report the results of the experiment designed, but not run, in Laubmeier et al. [30]. We minimized the error between time-series data from mesocosm experiments and a dynamic model, to determine underlying parameter values. We compared the fits of four alternative models (with versus without microhabitat use and non-consumptive predator-predator effects) to the observed dynamics of two aphid species and their predators. We found that the full model, with both microhabitat and non-consumptive predator-predator effects, performed the best across all our criteria. By comparing the four models and where they do and do not perform well, we discuss what we can learn about the importance of different mechanisms, the ecological implications of these mechanisms, and what traits we may be missing from the model. We discuss how our models can be used to predict novel scenarios, and how this can provide a test of our results. Finally, we discuss the value of iterative cycling between theory, data and experiment to hone current insights into how traits affect food-web dynamics.

The order of model performance suggests that, while both microhabitat use and non-trophic predator effects are important, microhabitat use is the more important of the two factors. A closer look however, tells us that this is probably only true for larger predators that are less affected by non-trophic predator interactions. Despite performing nearly as well as the full model in terms of model fit, the model with only microhabitat use had the most uncertainty associated with its estimates of *R*_*opt*_, particularly for *O. majusculus* and *Bembidion*. For these two predators, this model actually performed the worst of all models (Fig 4). *O. majusculus* and *Bembidion* are the smallest and most abundant predators, and therefore probably the most affected by non-trophic predator interactions, both from conspecifics and from larger predators. For intraguild prey, therefore, non-trophic predator effects likely play a similar, or potentially even larger role than overlap in microhabitat use. These results support the findings of Jonsson et al. [17], who found that added trophic complexity lead to weaker than expected trophic interaction strengths.

Our results give quantitative support to research highlighting the importance of microhabitat use as a factor driving community dynamics in arthropod communities (noted both in mesocosms [3, 17, 21, 51, 52] and in the field [53]). However, not all research examining the role of microhabitat use has found that it affects trophic interactions (e.g. [19]). This may be due to differences in the microhabitat or species involved, but may also be due to the way in which microhabitat overlap has been calculated. In many cases, very simple measures, such as a binary classification of microhabitat, are used, which may mask important variation in the way species use microhabitat. Here, we have introduced a quantitative metric that corrects previous metrics (e.g. [17, 30]) to accurately capture overlap in species’ use of microhabitat. We have shown that a) incorporating habitat overlap and non-trophic predator effects into a dynamic trophic interaction model does, in fact, improve our predictions of community dynamics, as well as b) demonstrating how to measure and incorporate these factors into a dynamic model of trophic interactions.

Habitat use and non-trophic predator interactions are both factors that can buffer the ‘negative effect’ of predation and competition and therefore enable coexistence [23, 52, 54]. However, this also means that if these factors are not considered, any predictions we make may be inaccurate. For example, when accounting for microhabitat use, *Bembidion* and *C. septempunctata* are predicted to have roughly similar *per capita* feeding rates on *R.padi* (40–50 individuals per day). However, despite having a much larger population size than *C. septempunctata* (40 vs 4 in single-predator treatments), *Bembidion* has a much smaller effect on *R.padi* populations. This is due to two factors: 1) the effect of non-trophic predator effects driving down the effective feeding rate of *Bembidion* (see S1 Fig) and 2) that despite similar overall overlap, *C. septempunctata* overlaps with all areas used by *R.padi*, whereas *Bembidion* overlaps with only 10% of the population. This gives *R.padi* a refuge from *Bembidion* predation where it can survive and reproduce, and prevents *Bembidion* from driving the population down as effectively as *C. septempunctata*. This is important; it means that few predator individuals may continue to have the same impact as many predator individuals. Similarly, in the presence of intraguild predators, intraguild prey often change their foraging behaviour or spatial distribution [55, 56], decreasing the combined effect of both predators [51, 55]. These mechanisms are particularly important when it comes to pest control; adding more predators as biocontrol agents may have less effect than anticipated [51]. Together, including both microhabitat use and non-trophic predator interactions in our model shows how predation effects are often not additive and can deviate from pairwise predictions, with important consequences.

By comparing parameter values across models, we can learn something not only about the mechanisms each model is predicting but also about traits or mechanisms that may be missing from our models. For example, *R*_*opt*_ values for *C. septempunctata* and *Bembidion* were consistently much higher than for *O. majusculus* and *Pardosa*, meaning that the beetles had higher attack rates on relatively smaller prey. This is likely because of differences in the way that beetles forage as compared to *Pardosa* (a spider) and *O. majusculus* (a bug) [57, 58]. *Bembidion* has higher final experimental population sizes than predicted by most models, suggesting *Bembidion* likely has traits, such as a tough cuticle [18], which decrease mortality rate from other predators but are not included in the model. These traits—foraging mode and defensive traits—are therefore likely candidates to be integrated into further iterations of trait-based models [8]. Adding the right traits may even remove the need for variable *R*_*opt*_ values, taking us closer to a fully trait-based model. Of course, adding extra traits comes with extra work associated with measuring them, and the optimal traits for a given study may vary (see [8] for a framework for selecting traits).

By building our models on species’ traits, we can make predictions for novel scenarios. We can incorporate species new to the community by measuring traits and then predicting what effect the new species will have on community dynamics. While empirically implementing such an experiment would have exceeded our current resources, we demonstrate how to do so by predicting the population dynamics of a hypothetical new prey species with each predator species in Fig 4. Applying the model to new predator and/or prey species is also a way to test the generality of these models, by determining which model provides the most accurate prediction. Most importantly, these predictions concern quantities which can explicitly be tested in future experiments. Thus, the dynamics observed in our mesocosms provide the first step for distinguishing between models. But, since we can i) make predictions using the models and ii) explicitly test those predictions, the ultimate test of the validity of our models should be based on further, iterating cycling between predictions and further experiments. A model built on traits also allows us to predict how interactions and dynamics will respond to a change in traits or the environment [8]. For example, if predator and prey change their microhabitat use to overlap more or less (as may be caused, for example, by differences in landscape [35] or loss of habitat e.g. [59]), then a model including microhabitat use will have vastly different predictions from one not including microhabitat use. As we begin to build our library of trait-based models and our understanding of where and how different traits affect different parts of the predation process, we will increase our capacity to predict and manage the dynamics of ecological communities in diverse and changing ecosystems [8].

We used least squares minimization [38, 60] to fit our models. This method, as many others, is designed to find the parameter values that give the best fit possible to the data. This does not, however, mean that those parameter values are true, and we must be careful not to conflate a well-fitting model with causal evidence for the importance of the modelled mechanism. We tried to protect against such flawed inference in three ways here. First, we used data from a previous experiment, to run pre-experimental simulations and determine the optimal sampling method, ensuring we would have the greatest capacity to distinguish between models. This is presented in [30]. Second, we compared the fit of four different models across a range of criteria. This meant i) we could compare multiple models and not limit ourselves to a single model and ii) that we evaluated the models not only on their fit to the data but also in their realism. Third, we restricted the parameters to fall within realistic bounds as determined from the literature (S1 Table in S1 File). This prevented the parameters from taking on unrealistic values to fit the data in models that did not account for important mechanisms. In a preliminary analysis (not shown here) we found that when we did not impose realistic limits on the parameter values all models had very similar predictions (i.e. JLS cost criterion and predictions of aphid dynamics), but did so by having very unrealistic parameter values in many cases. We do note that, here, fitted *R*_*opt*_ values for *C. septempunctata* and *Bembidion* consistently were at or near their upper boundary (Table 1). These boundary values were determined from interactions reported in the literature, which include the most common prey items for these predators and should, therefore, include plausible *R*_*opt*_ values. We therefore suspect that the model hits the upper boundary because there are traits not included in the model, which are also important for the interactions. As an alternative, the ranges that we set for these *R*_*opt*_ values might not have been wide enough, despite their foundation in the literature. The latter would however imply that, in the wild, these predators are basing much of their feeding on significantly suboptimal prey (from a foraging success point of view). To resolve the issue, predator-specific *R*_*opt*_ values would be determined by targeted experiments so that we could be confident that these values are correct, and that if the model is hitting the boundaries it is because the model needs to be adjusted. Thus, such iterative cycling between theory, existing information, experiment, and data as used here, will be essential to further increase our understanding of how traits affect food-web dynamics. While technically feasible, such work calls for substantial investment of time, and therefore fell beyond the remits of the current study.

## Conclusions

In this study, we tested an approach explicitly developed in Laubmeier et al. [30]. We developed the ATN model to include, in addition to body size, microhabitat overlap and non-trophic predator-predator interactions. By comparing four models, we found that both overlap in microhabitat use and non-trophic predator-predator interactions improved our ability to explain aphid and predator dynamics. While body size has been successful in predicting structure and dynamics of many trophic networks, it does not explain everything, and our results are a step toward creating trait-based models effective in a wider range of scenarios. Importantly, we demonstrated how we can leverage pre-existing data and the results of other studies to most effectively develop these models.

## Supporting information

S1 File **S1.1** Experimental design, **S1.2** Model fitting, **S1.3** Parameter units, **S1.4** Sensitivity equations for parameter confidence intervals. Includes **S1 Table** listing bounds imposed on initial abundances, **S2 Table** bounds imposed on parameter values, and **S3 Table** meaning and units of each parameter.
(PDF)

S1 Fig Predator feeding rates in the presence and absence of conspecific predator individuals. (PDF)
