# Supplementary material for: Beyond body size—new traits for new heights in trait-based modelling of predator-prey dynamics
Source: PLoS One. 2022 Jul 21;17(7):e0251896. doi: 10.1371/journal.pone.0251896 (PMC9302725; doi:10.1371/journal.pone.0251896)
Supplement: S1 File — S1.1 Experimental design, S1.2 Model fitting, S1.3 Parameter units, S1.4 Sensitivity equations for parameter confidence intervals. Includes S1 Table listing bounds imposed on initial abundances, S2 Table bounds imposed on parameter values, and S3 Table meaning and units of each parameter. (PDF) [file pone.0251896.s001.pdf]

Supplementary methods for:

Beyond body size — new traits for new heights in trait-based modelling of predator-prey dynamics

Kate L. Wootton, Alva Curtsdotter,
Tomas Jonsson, H.T. Banks, Riccardo Bommarco, Tomas Roslin and Amanda N. Laubmeier

S1 Supplementary methods

S1.1 Experimental design

Orius majusculus were purchased from Lindesro AB at the beginning of each run. Other predators were captured in the field and stored at 4 °C and kept humid for at least 48 hours prior to being added to the experiment. Aphids were obtained from a colony maintained in the lab.

The plants in the cages were grown in a seed- and insect-free peat-perlite-sand mixture (70-25-5, P-jord, Hasselfors Garden Ltd.). Two rows of 10 fava beans (20 total) were sown 12 days before the aphids were added. Four days later, 3 rows of 15 barley seeds (45 total) were planted. 8 days after the barley was planted, the mesocosms were thinned to make sure there were exactly 15 plants per row of barley and 10 per row of beans. A thin layer of white sand was added over the soil to improve the observation of predators. The mesh cage had an area of 60x40 cm and mesh size 0.5x0.5 mm. Plants were watered as required throughout the experiment.

Aphids were allowed to colonize plants for 24 hours, after which the Petri dishes were checked. Dead aphids were replaced with new adults. After another 24 hours, predators were introduced to the microcosms and the eight-day experiment began.

Each predator-prey combination (33 in total) was replicated six times, leading to a total of 196 mesocosms to run. Due to space and time limitations, this required that they be split into 4 runs, such that up to 50 mesocosms were run at one time. We utilized two greenhouses and half of the 50 mesocosms were assigned to each greenhouse. Treatments were split such that each treatment appeared in every run, with the remaining replicates assigned at random. No two treatments were run in the same greenhouse at the same time.

S1.2 Model fitting

Numerical results were obtained using MATLAB version R2020a (9.8.0.1417392) on a linux machine (5.4.0-89-generic). The code is available with the raw data in the linked repository. Model solutions were obtained using `ode45` for speed, but we enforced non-negative constraints for all populations, which we observed was sufficient to match accuracy of more intensive solvers. Individual iterations of the minimizing function `fmincon` had step size tolerance of 10^{-10} and optimality tolerance of 10^{-6}. This algorithm permits constraints on model parameters, which we determined from

experimental observations, available literature, and numerical experiments. For aphid population parameters (r_i and initial abundance), which remained constant across models, we report the parameters in Table S1 and discuss the source of constraints in the table caption. For ATN model parameters, we report the values in Table S2 and discuss in greater detail through the following paragraphs.

Table S1: Bounds imposed on parameter values fit to control data. The initial abundance bounds are informed by experimental observations and the bounds for r_i did not affect estimation (wider parameter bounds were used, then reduced to around the consistently estimated values of 0.35).

Aphid	Treatment	Parameter	min	max
R. padi	alone	initial abundance	175	475
A. pisum	alone	initial abundance	125	775
R. padi	combined	initial abundance	50	225
A. pisum	combined	initial abundance	50	275
All	All	r_i	0.25	0.45

Table S2: Bounds imposed on parameter values during model fitting. When multiple bounds are listed, we experimented with multiple ranges during model fitting to ascertain the source of unbounded a_0 estimates (see discussion below for more details).

Predator	Parameter	min	max
Coccinella	R_{opt}	5	250
Pardosa	R_{opt}	0.05	100
Orius	R_{opt}	0.05	175
Bembidion	R_{opt}	30	200
All	h_0	4×10^{-11}	1.3
	b_0	5	20
	ϕ	0.1	1.5
		0.5	1.5
		0.05	0.5
	a_0	1	10
		1	25
		1*	300*

R_{opt} values for *C. septempunctata*, *Pardosa* and *Orius* were calculated from functional responses recorded in the database in Uiterwaal et al. (2018). For each predator, we took all records for that predator, calculated the predator-prey body mass ratio (PPBMR) for each record, and then used the range of PPBMR as the bounds of R_{opt} for that predator, rounded outwards (i.e. down for lower bounds and up for upper bounds) to the nearest round numbers. There were no records for *R. padi* as a prey item of *C. septempunctata* in the dataset by Uiterwaal et al. (2018), but we knew from our own research and others (e.g. Jonsson et al., 2018; Roubinet et al., 2018) that *C. septempunctata*

is definitely a predator of *R. padi*, so we extended the $R_{opt,C}$ range to 250 to include the *R. padi* (which has a PPBMR of 239 with *C. septempunctata*). By including all interactions recorded for the predator, across a range of prey, the *optimal* PPBMR (R_{opt}) should be included within the range of *possible* PPBMRs. There were no records for *Bembidion* in Uiterwaal et al. (2018), so we scoured the literature for records of *Bembidion*s prey (Mitchell, 1963; Roubinet et al., 2018; Hering and Plachter, 1997) and measurements of the prey's body size (Langevelde et al., 2020; Füreder and Niedrist, 2020; Ortega-Jiménez et al., 2016) and applied the same process to calculate $R_{opt,B}$ as for the other predators. To calculate the range for h_0, we took all handling times recorded in Uiterwaal et al. (2018) for our predators and solved Eq. (2) for h_0 for each record, then took the minimum and maximum calculated h_0 values as our range.

For remaining model parameters, we did not have reliable information to inform the bounds for minimization. Instead, we experimented with bounds to determine bounds which did not impact the results of the minimization. The bounds for b_0 reliably did not impact its estimated value. The bounds for ϕ and a_0 were more sensitive, and some model formulations resulted in minimizations at the boundary of permitted parameter values. We used several different bounds to determine appropriate ranges, as indicated in Table S2. For a_0 bounds with a "*" we noted that the minimization frequently took a_0 to an upper bound; we attempted subsets of size 10, 25, and 50 within this range to test if the movement towards an upper bound was reasonable. After determining that no upper bound up to 300 could not be reached (and minimal impact on model solutions for very large a_0), we returned to the arbitrary choice of 25 and used the bounds $1 \leq a_0 \leq 25$. This choice affects models without microhabitat. At the same time, we observed that when a_0 increased to its upper bound, ϕ could reduce towards lower bounds. We experimented with the three listed ranges alongside the different ranges for a_0. The final range for all ϕ fell between $0.05 \leq \phi 1.5$, since results could fall to either extremes (high or low values) depending on the value of a_0. The `multistart` algorithm repeated for 25 iterations in the final results; in selecting this number, we conducted experiments with up to 100 iterations over the widest and most narrow parameter ranges for a_0 and ϕ, without observing a change in the reported "global" minimization (or a tendency to the upper or lower bound, in cases where the range omitted the minimizing value).

S1.3 Parameter units

Units for quantities in our model are given in Table S3. Quantities use the same notation as the model in Section 2 of the main manuscript, with the exception of F_{jm}, which denotes the functional response for species j in microhabitat m as in Section S1.4 of the Supplementary Material.

The unit "cage" refers to the size of a single mesocosm, which was consistent in our experiments. However, the equivalent density-based unit would be an area or volume, such as m^2 or m^3. Quantities A_m were determined relative to the baseline size of one of the defined microhabitats (the ground). The unit "habitat" refers to this area; otherwise, this could also use a measure of area such as m^2. In measuring p_{im}, we computed the ratio of individuals in microhabitat m (ind_i/microhabitat) to individuals in the mesocosm (ind_i/cage). This led to the units $\frac{\text{ind}_i/\text{microhabitat}}{\text{ind}_i/\text{cage}}$, or cage/microhabitat.The remaining quantities are measured in terms of standard quantities: individuals of species i ("ind_i"), days ("day"), and micrograms ("mg"). Most units follow directly from these measurements, or unit balancing in the model equations.

Table S3: Units of measurement for model quantities, and their meaning. The abbreviation "ind_i" is for "individuals of species i." Quantities with units given by "–" are dimensionless.

Quantity	Meaning	Units of measurement
N_i	population of species i	ind_i/cage
t	time	day
W_i	mass of individual of species i	mg
p_{im}	likelihood of i residing in microhabitat m	cage/microhabitat
A_m	area of microhabitat m	microhabitat/cage
r_i	growth of species i	1/day
a_{ij}	attack of j on i	$\frac{1}{\text{ind}_i}\frac{1}{\text{day}}\frac{1}{\text{microhabitat}}$
F_{jm}	functional response of j in microhabitat m	ind_j
h_{kj}	handling time of j consuming k	ind_j/day
t_0	predator avoidance	ind_j/day
R_{opt}	optimal predator-prey body mass ratio	–
ϕ	scaling power for attack rate	–
a_0	scaling constant for attack rate	$\frac{1}{\text{ind}_i}\frac{1}{\text{day}}\frac{1}{\text{microhabitat}}\frac{1}{\text{mg}^{1/2}}$
h_0	scaling constant for handling time	ind_j/day

S1.4 Sensitivity equations for parameter confidence intervals

In order to compute confidence intervals using sensitivity matrices, we computed the sensitivity equations (partial derivatives of observed data with respect to model parameters) for our model. For $f_i = \frac{dN_i}{dt}$, the partial derivative of f_i with respect to the number of individuals of any species (denoted N_d) is

$$\frac{\partial f_i}{\partial N_d} = r_i \frac{\partial N_i}{\partial N_d} - \sum_j \sum_h \frac{p_{ih} p_{jh} a_{ij}}{F_j h} \left(\frac{\partial N_i}{\partial N_d} N_j + N_i \frac{\partial N_j}{\partial N_d} - \frac{N_i N_j}{F_{jh}} \frac{\partial F_{jh}}{N_d} \right),$$

where

$$F_{jh} = A_h + \sum_k p_{kh} a_{kj} h_{kj} N_k + t_0 \sum_l p_{lh} a_{jl} N_l$$

and

$$\frac{\partial F_{jh}}{\partial N_d} = \sum_k p_{kh} a_{kj} h_{kj} \frac{\partial N_k}{\partial N_d} + t_0 \sum_l p_{lh} a_{jl} \frac{\partial N_l}{\partial N_d}.$$

The partial derivative of f_i with respect to a model parameter θ is

$$\frac{\partial f_i}{\partial \theta} = -\sum_j \sum_h \frac{p_{ih} p_{jh} N_i N_j}{F_{jh}} \left(\frac{\partial a_{ij}}{\partial \theta} - \frac{a_{ij}}{F_{jh}} \frac{\partial F_{jh}}{\partial \theta} \right),$$

where for $\theta_a = a_0, R_{opt,j}, \phi$ we have

$$\frac{\partial F_{jh}}{\partial \theta_a} = \sum_k p_{kh} h_{kj} N_k \frac{\partial a_{kj}}{\partial \theta_a} + t0 \sum_l p_{lh} N_l \frac{\partial a_{jl}}{\partial \theta_a},$$

$$\frac{\partial F_{jh}}{\partial h_0} = \sum_k p_{kh} h_{kj} N_k \frac{h_{kj}}{h_0},$$

$$\frac{\partial F_{jh}}{t_0} = \sum_l p_{lh} a_{jl} N_l.$$

And for the different θ_a, the partial derivatives of a_{ij} with respect to θ_a are

$$\frac{\partial a_{ij}}{\partial a_0} = a_{ij}/a_0$$

$$\frac{\partial a_{ij}}{R_{opt,j}} = a_{ij} \frac{\phi}{R_{opt,j}} \left(\frac{W_j/W_i}{R_{opt,j}} - 1 \right)$$

$$\frac{\partial a_{ij}}{\partial \phi} = a_{ij} \ln \left(\frac{W_j/W_i}{R_{opt,j}} e^{1 - \frac{W_j/W_i}{R_{opt,j}}} \right)$$

References

Füreder, L. and Niedrist, G. H. (2020). Glacial stream ecology: Structural and functional assets. *Water (Switzerland)*, 12(2).

Hering, D. and Plachter, H. (1997). Riparian ground beetles (Coeloptera , Carabidae) preying on aquatic invertebrates : a feeding strategy in alpine floodplains. *Oecologia*, 111:261–270.

Jonsson, T., Kaartinen, R., Jonsson, M., and Bommarco, R. (2018). Predictive power of food web models based on body size decreases with trophic complexity. *Ecology Letters*, 21(5):702–712.

Langevelde, F. V., Comor, V., De Bie, S., Prins, H. H. T., and Thakur, M. P. (2020). Disturbance regulates the density-body-mass relationship of soil fauna. *Ecological Applications*, 20(1):e02019.

Mitchell, B. (1963). Ecology of Two Carabid Beetles, Bembidion lampros (Herbst) and Trechus quadristriatus (Schrank). *The Journal of Animal Ecology*, 32(3):377.

Ortega-Jiménez, V. M., Arriaga-Ramirez, S., and Dudley, R. (2016). Meniscus ascent by thrips (Thysanoptera). *Biology Letters*, 12(9).

Roubinet, E., Jonsson, T., Malsher, G., Staudacher, K., Traugott, M., Ekbom, B., and Jonsson, M. (2018). High redundancy as well as complementary prey choice characterize generalist predator food webs in agroecosystems. *Scientific Reports*, 8(1):1–10.

Uiterwaal, S. F., Lagerstrom, I. T., Lyon, S. R., and DeLong, J. P. (2018). Data paper: FoRAGE (Functional Responses from Around the Globe in all Ecosystems) database: a compilation of functional responses for consumers and parasitoids. *bioRxiv*, pages 1–43.
