# Supplementary material for: Beyond body size—new traits for new heights in trait-based modelling of predator-prey dynamics
Source: PLoS One. 2022 Jul 21;17(7):e0251896. doi: 10.1371/journal.pone.0251896 (PMC9302725; doi:10.1371/journal.pone.0251896)
Supplement: S1 Fig — (PDF) [file pone.0251896.s002.pdf]

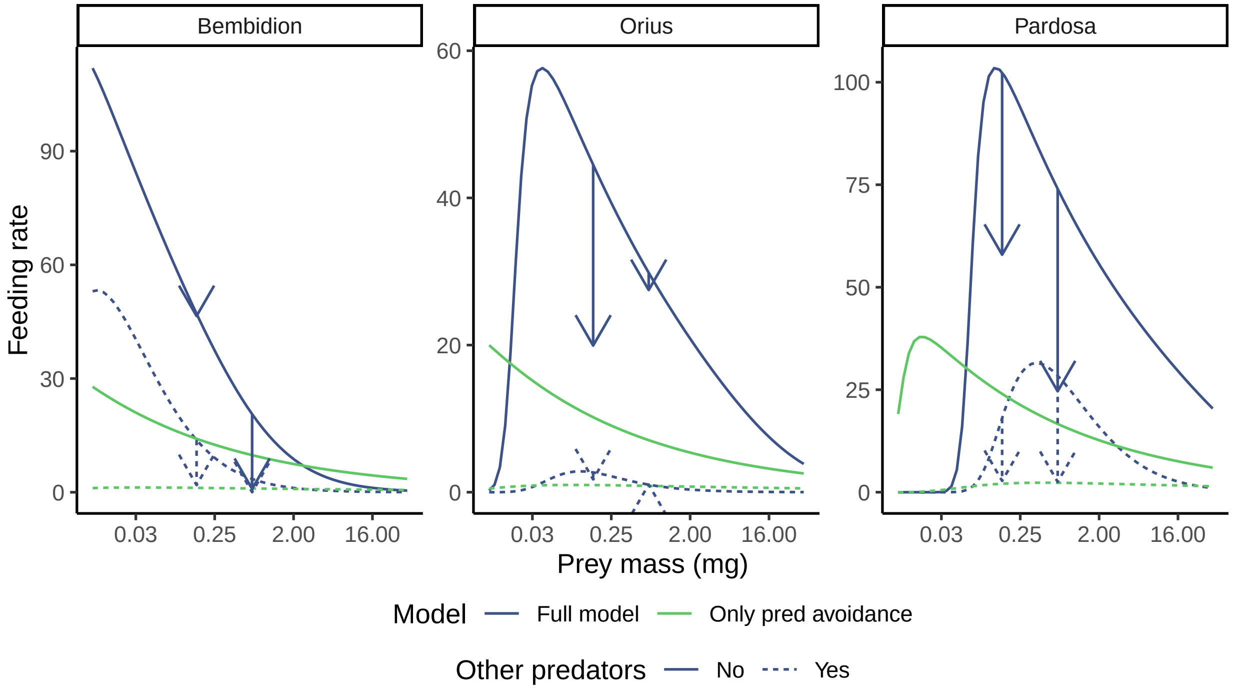

Figure S1: Comparison of individual predators' feeding rates in the presence (dashed line) or absence (solid line) of conspecific predator individuals for models that include non-trophic predator interactions (t_0). Model predictions for an individual predator's feeding rate (number of prey consumed per predator per day, y axis) on prey of different body sizes (x axis). Curves show the "habitat-free" feeding rate, i.e. assuming all species use all microhabitats in proportion to their area, while vertical arrows show the difference in feeding rate in the full model when accounting for observed microhabitat preferences of the predator and *R.padi* (mass=0.155mg) and *A.pisum* (mass=0.67mg). Line color corresponds to different models. Solid lines show the instantaneous feeding rate with a population of 250 aphids and in the *absence* of any other predator individuals (i.e. not accounting for non-trophic predator effects), while dashed lines show an individual predator's instantaneous feeding rate when in the presence of 20 *Bembidion*, 20 *O. majusculus* or 10 *Pardosa* conspecific individuals respectively. Observe that, for *Bembidion*, accounting for microhabitat use of *R.padi* does not substantially change the predictions of models with microhabitat (i.e. the arrows sit on the curve). This is because the overlap of *Bembidion* with *R.padi* works out to be almost the same as if they used all microhabitats in proportion to the size of the microhabitat. Note that we do not include *C. septempunctata* because we did not include feeding interactions with the other predators. Note the varying scales of the y-axis.
